# Biomarkers to safely discharge head trauma patients in the COVID-19 pandemic era

**DOI:** 10.2217/bmm-2021-0008

**Published:** 2021-03-05

**Authors:** George A Alexiou, Georgios D Lianos, Eleftherios-Spyridon Alexiou, Spyridon Voulgaris

**Affiliations:** ^1^Department of Neurosurgery, University Hospital of Ioannina, Ioannina, Greece; ^2^Department of Surgery, University Hospital of Ioannina, Ioannina, Greece

**Keywords:** COVID-19, emergency department, general surgeon, head trauma, neurosurgeon, pandemic era

SARS-CoV-2 or COVID-19 caused the pandemic that was declared by the WHO on 11 March 2020. The current coronavirus pandemic resulted in overwhelming numbers of patients presenting to the emergency department and unprecedented care requirements. Health systems in several countries were on the verge of collapse, not to mention the economic downturn. The mandatory quarantine and restrictions in daily living, implemented by several countries, have been proven to reduce traumatic brain injury (TBI) cases significantly [1]. However, in relation to the mechanism of injury, a relative increase in low-impact falls at home was seen [1].

Mild TBI is the most common form of TBI, accounting for 70–90% of all brain injuries. The estimated incidence is about 600 cases per 100,000 of the population, being more common in males and young adults [2]. In patients presenting with a Glasgow Coma Scale score of 15, about 5% of CT scans have pathological findings, whereas in patients over 65 years of age, 11–21% may have findings consistent with intracranial trauma. Haydel *et al.* reported that 17% of adults over 60 years of age with a normal Glasgow Coma Scale at admission had a positive head CT [3]. Neurosurgical intervention is required in approximately 1% of mild TBI patients. The epidemiology pattern of TBI has changed in recent years, with falls in the elderly increasing significantly, mainly in high-income countries [4]. In children, 640,000 visits per year to the emergency department have been reported in the USA. Falls and being struck by an object were the most common causes in the younger age group, whereas motor accidents and falls were the most frequent in the older groups [5].

Indiscriminate use of CT is associated with high cost and risk of radiation exposure, especially in children. A study that evaluated cancer risk in 680,000 children and adolescents that were exposed to a diagnostic CT found a 24% higher cancer incidence in exposed people [6]. MRI in the acute setting has also been investigated and is currently used in some institutes [7]. Roguski *et al.* evaluated 35 children with TBI that underwent both CT and MRI examinations within a 5-day period, and found that MRI can be a useful alternative to CT. Nevertheless, MRI missed skull fractures in five out of 13 cases [8]. Thus, physicians must decide which patient are in need of neuroimaging, which should be observed, and which can be discharged home. Several prediction rules to identify patients requiring a CT scan have been developed, however usage in clinical practice is variable or requires external validation.

During a pandemic, the identification of patients with mild TBI in need of CT and possible hospitalization is of paramount importance. First, the detection of patients in need of hospitalization would help minimize long waiting hours in the emergency department, in which there is a risk of virus transmission [9]. This would facilitate the transfer of the patient to a hospital capable of providing CT scans or neurosurgical intervention. Furthermore, some may choose to avoid emergency care, which may in turn delay treatment for new and concerning symptoms.

The mechanism by which these biomarkers are transported outside the brain is still under investigation. Several biomarkers in blood, corticospinal fluid, saliva or urine have been investigated in this regard [10]. TBI is known to cause blood–brain barrier disruption, however, this might not be the sole mechanism. In a murine TBI model it was reported that the glymphatic pathway transports biomarkers to the blood via the cervical lymphatics [11]. S100 astroglial calcium-binding protein B (S100B) is the most well-studied biomarker and has been evaluated within 6 h postinjury. A cut-off value of 0.10 μg/l has been used in the majority of studies with a sensitivity ranging from 72 to 100%. The specificity is low, in the range of 30% in most studies [12]. Ubiquitin C-terminal hydrolase-L1 (UCH-L1) is found in neurons and glial fibrillary acidic protein (GFAP) is an astroglial biomarker. Both have been evaluated for TBI detection. For UCH-L1, a cut-off value of 0.18 ng/ml yielded a 100% sensitivity, 47% specificity and a negative predictive value of 100%; whereas for GFAP, a cut-off value of 0.067 ng/ml showed 100% sensitivity and 55% specificity [13,14]. GFAP was superior to S100B in detecting intracranial lesions. In contrast to GFAP, S100B levels were affected by the presence of extracranial fractures [14]. A recent prospective, multicentre observational study evaluated the combination of UCH-L1 and GFAP in mild and moderate TBI patients within 12 h of injury. The sensitivity was found to be 97.6% and the negative predictive value 99.6%, and the first US FDA-approved tool was established [15]. Hyperglycemia after TBI is a known event. A study on pediatric TBI reported that serum glucose levels greater than or equal to 300 mg/dl at admission was consistently associated with death [16]. In a study that included 159 patients with mild TBI, a cut-off value of 120 mg/dl discriminated patients with positive CT findings with a sensitivity of 74.4% and a specificity of 90.7%. All patients with serum glucose levels greater than 128 mg/dl had an abnormal CT [17]. Elevated serum glucose levels were also found in patients that developed coagulopathy, which is associated with increased morbidity and mortality [18]. Neutrophil-to-lymphocytes ratio (NLR) has also been evaluated as an inexpensive biomarker for predicting the need for CT in mild TBI. In a study of 130 patients with mild TBI that underwent CT, 74 patients had abnormal CT findings. Patients with positive CT findings had 6.4 ± 5.3 NLR levels and patients with a negative CT had 3.6 ± 2.4 NLR levels, a statistically significant difference. A cut-off value of 2.5 was found to have 78.1% sensitivity and 63% specificity for detecting patients with abnormal CT findings [19].

One important issue in COVID-19 patients is the increased levels of GFAP, neurofilament light polypeptide (NfL) and tau in both cerebrospinal fluid and blood [20,21]. In a study of 47 patients with mild, moderate or severe COVID-19, GFAP and NfL were measured in plasma at two time points. The results showed that patients with severe disease had significantly higher serum concentrations of GFAP and NfL than the control group, and GFAP was also elevated in patients with moderate disease [20]. Thus, in the case of brain injury in COVID-19 patients, there is a need for careful interpretation of these biomarkers. Additionally, transfer of COVID-19 patients to neuroimaging is not always straightforward, has several difficulties and might need extra preparation; thus, biomarkers to detect patients in need of CT are important. Of note, GFAP has also been found to be elevated in cases of Alzheimer’s disease or tuberculous meningitis [21].

## Conclusion

The identification and use of biomarkers to predict the need for CT in mild TBI patients is of paramount importance. These biomarkers should be widely available and of low cost, especially during a pandemic, when available resources might be limited. In the COVID-19 pandemic, TBI biomarkers would result in more efficient management of non-COVID TBI patients. In patients infected with SARS-CoV-2 presenting with TBI, the careful interpretation of certain biomarkers is important. More studies are needed to investigate the value and cost–effectiveness of a single or combination of biomarkers for both COVID and non-COVID TBI patients.

## References

[1] Pinggera D, Klein B, Thomé C, Grassner L. The influence of the COVID-19 pandemic on traumatic brain injuries in Tyrol: experiences from a state under lockdown. Eur. J. Trauma Emerg. Surg. 22, 1–6 (2020). 10.1007/s00068-020-01445-7 PMC7374069 32699916

[2] Cassidy JD, Carroll LJ, Peloso PM WHO collaborating Centre Task Force on mild traumatic brain injury. incidence, risk factors and prevention of mild traumatic brain injury: results of the WHO collaborating centre task force on mild traumatic brain injury. J. Rehabil. Med. 43(Suppl.), 28–60 (2004). 10.1080/16501960410023732 15083870

[3] Haydel MJ, Preston CA, Mills TJ, Luber S, Blaudeau E, DeBlieux PM. Indications for computed tomography in patients with minor head injury. N. Engl. J. Med. 343(2), 100–105 (2000). 10891517 10.1056/NEJM200007133430204

[4] Maas AIR, Menon DK, Adelson PD Traumatic brain injury: integrated approaches to improve prevention, clinical care, and research. Lancet Neurol. 16(12), 987–1048 (2017). 29122524 10.1016/S1474-4422(17)30371-X

[5] The Lancet. The burden of traumatic brain injury in children. Lancet 391(10123), 813 (2018). 29508730 10.1016/S0140-6736(18)30547-6

[6] Mathews JD, Forsythe AV, Brady Z Cancer risk in 680,000 people exposed to computed tomography scans in childhood or adolescence: data linkage study of 11 million Australians. BMJ 346, f2360 (2013). 23694687 10.1136/bmj.f2360 PMC3660619

[7] Argyropoulou MI, Alexiou GA, Xydis VG Pediatric minor head injury imaging practices: results from an ESPR survey. Neuroradiology 62(2), 251–255 (2020). 31828360 10.1007/s00234-019-02326-6

[8] Roguski M, Morel B, Sweeney M Magnetic resonance imaging as an alternative to computed tomography in select patients with traumatic brain injury: a retrospective comparison. J. Neurosurg. Pediatr. 15(5), 529–534 (2015). 25700122 10.3171/2014.10.PEDS14128

[9] Esteve-Esteve M, Bautista-Rentero D, Zanón-Viguer V. Risk of influenza transmission in a hospital emergency department during the week of highest incidence. Emergencias 30(1), 7–13 (2018). 29437304

[10] Alexiou GA, Lianos GD, Sotiropoulos A, Voulgaris S. Novel biomarkers may aid the decision for CT scan in emergency settings in mild head trauma. Biomark. Med. 13(13), 1055–1057 (2019). 31468996 10.2217/bmm-2019-0277

[11] Plog BA, Dashnaw ML, Hitomi E Biomarkers of traumatic injury are transported from brain to blood via the glymphatic system. J. Neurosci. 35(2), 518–526 (2015). 25589747 10.1523/JNEUROSCI.3742-14.2015 PMC4293408

[12] Heidari K, Vafaee A, Rastekenari AM S100B protein as a screening tool for computed tomography findings after mild traumatic brain injury: systematic review and meta-analysis. Brain Inj. 29(10), 1146–1157 (2015). 26067622 10.3109/02699052.2015.1037349

[13] Papa L, Mittal MK, Ramirez J Neuronal biomarker ubiquitin C-terminal hydrolase detects traumatic intracranial lesions on computed tomography in children and youth with mild traumatic brain injury. J. Neurotrauma 34(13), 2132–2140 (2017). 28158951 10.1089/neu.2016.4806 PMC5510668

[14] Papa L, Silvestri S, Brophy GM GFAP out-performs S100β in detecting traumatic intracranial lesions on computed tomography in trauma patients with mild traumatic brain injury and those with extracranial lesions. J. Neurotrauma 31(22), 1815–1822 (2014). 24903744 10.1089/neu.2013.3245 PMC4224051

[15] Bazarian JJ, Biberthaler P, Welch RD Serum GFAP and UCH-L1 for prediction of absence of intracranial injuries on head CT (ALERT-TBI): a multicentre observational study. Lancet Neurol. 17(9), 782–789 (2018). 30054151 10.1016/S1474-4422(18)30231-X

[16] Cochran A, Scaife ER, Hansen KW, Downey EC. Hyperglycemia and outcomes from pediatric traumatic brain injury. J. Trauma 55(6), 1035–1038 (2003). 14676647 10.1097/01.TA.0000031175.96507.48

[17] Alexiou GA, Sotiropoulos A, Lianos GD Blood glucose levels may aid the decision for CT scan in minor head trauma. Dis. Markers 2019, 1065254 (2019). 31093304 10.1155/2019/1065254 PMC6481103

[18] Alexiou GA, Lianos G, Fotakopoulos G, Michos E, Pachatouridis D, Voulgaris S. Admission glucose and coagulopathy occurrence in patients with traumatic brain injury. Brain Inj. 28(4), 438–441 (2014). 24564221 10.3109/02699052.2014.888769

[19] Alexiou GA, Lianos GD, Tzima A Neutrophil to lymphocyte ratio as a predictive biomarker for computed tomography scan use in mild traumatic brain injury. Biomark. Med. 14(12), 1085–1090 (2020). 32969245 10.2217/bmm-2020-0150

[20] Kanberg N, Ashton NJ, Andersson LM Neurochemical evidence of astrocytic and neuronal injury commonly found in COVID-19. Neurology 95, e1754–e1759 (2020). 32546655 10.1212/WNL.0000000000010111

[21] DeKosky ST, Kochanek PM, Valadka AB Blood biomarkers for detection of brain injury in COVID-19 patients. J. Neurotrauma 38(1), 1–43 (2020). 33115334 10.1089/neu.2020.7332 PMC7757533

